# Gene ontology enrichment analysis of congenital diaphragmatic hernia-associated genes

**DOI:** 10.1038/s41390-018-0192-8

**Published:** 2018-09-25

**Authors:** Timothy R. A. Dalmer, Robin D. Clugston

**Affiliations:** Department of Physiology, and Women and Children’s Health Research Institute, University of Alberta, Edmonton, AB Canada

## Abstract

Congenital diaphragmatic hernia (CDH) is a commonly occurring major congenital anomaly with a profound impact on neonatal mortality. The etiology of CDH is poorly understood and is complicated by multiple clinical presentations, reflecting the location and type of diaphragm defect. With the increased power of genetic screening, more genes are being associated with CDH, creating a knowledge gap between CDH-associated genes and their contribution to diaphragm embryogenesis. Our goal was to investigate CDH-associated genes and identify common pathways that may lead to abnormal diaphragm development. A comprehensive list of CDH-associated genes was identified from the literature and categorized according to multiple factors, including type of CDH. We undertook a large-scale gene function analysis using gene ontology to identify significantly enriched biological pathways and molecular functions associated with our gene set. We identified 218 CDH-associated genes. Our gene ontology analysis showed that genes representing distinct biological pathways are significantly enriched in relation to different clinical presentations of CDH. This includes retinoic acid signaling in Bochdalek CDH, myogenesis in diaphragm eventration, and angiogenesis in central tendon defects. We have identified unique genotype–phenotype relationships highlighting the major genetic drivers of the different types of CDH.

## Introduction

Congenital diaphragmatic hernia (CDH) is a severe developmental defect associated with significant neonatal mortality and morbidity. In general, incomplete development of the diaphragm allows the abdominal contents to invade the thoracic cavity, resulting in compression of the developing lungs.^1^ Lung abnormalities caused by CDH include pulmonary hypoplasia and hypertension, and mean that newborns with CDH have a compromised ability to breathe, often presenting as neonatal emergencies.^1^ CDH is one of the most commonly occurring major congenital anomalies, which affects approximately 1 in 3000 live births with no effective prenatal intervention, and a mortality rate reported between 30 and 80%, depending on multiple factors.^2,3^ Despite its high incidence and deleterious impact on neonatal health, the etiology of CDH remains poorly understood. It is thought that both genetic and environmental factors contribute to CDH’s etiology, with 10–20% of cases having an identifiable genetic cause.^3–5^ Recently, advances in genetic testing and DNA sequencing (including whole-genome sequencing and whole-exome sequencing), have led to an increase in the number of candidate disease-causing genes that have been associated with CDH.^6–13^ As emphasized in a recent review by Kardon and colleagues, as the number of CDH-associated genes grows, the identification of “common downstream pathways” will be essential to better understand how CDH develops, and for the identification of common therapeutic targets that could be used to promote diaphragm growth.^14^ In accord with this gap in the literature, one of our laboratory’s goals is to improve our understanding of the genetic etiology of CDH.

In the context of this paper, it is important to emphasize that the term CDH can be used to describe several different clinical presentations that depend on the position and type of diaphragm defect.^15^ The most common and clinically relevant type of CDH is a Bochdalek hernia, which accounts for ~70% of cases and is characterized by a hole located in the postero-lateral region of the diaphragm.^15,16^ Morgagni (retrosternal) hernias (10–27% of cases) are characterized by hernias found in the anterior portion of the diaphragm, adjacent to the sternum, occurring through the foramen of Morgagni.^15,16^ Diaphragm eventration is characterized by incomplete formation of the diaphragm musculature. This can occur in different portions of the diaphragm and does not include communication between the peritoneal and thoracic cavities; rather the weakened diaphragm can be pushed upwards by the underlying organs, compressing the developing lungs. While diaphragm eventration is certainly a less common phenotype of CDH, difficulty arises in estimating the exact frequency of these defects as they are often misdiagnosed, thus there is a lack of an accurate estimated prevalence of this rare type of CDH.^1,15,16^ The final commonly recognized type of CDH are central tendon defects (2–3% of cases), which involve incomplete formation of the diaphragm’s central tendon.^15,16^ The classification of these different phenotypes of diaphragm defects is important in the context of the complex genetic etiology of CDH because we believe that each type of CDH has a unique genetic underpinning.

The goal of this study was to analyze a comprehensive list of CDH-associated genes using gene ontology to identify common pathways that may lead to abnormal diaphragm development. Based on the identification of 218 CDH-associated genes, we have revealed several common pathways that contribute to the development of different CDH phenotypes. As our knowledge about the genetic etiology of CDH grows, we will be better positioned to understand how different types of diaphragm defects arise, providing crucial information for the prenatal diagnosis of CDH, estimating prognosis, and deciding on possible treatment strategies.

## Methods

### CDH-associated genes data acquisition

We assembled an exhaustive list of CDH-associated genes through a comprehensive search of research articles published in the MEDLINE bibliographic database using the PubMed search engine (https://www.ncbi.nlm.nih.gov/pubmed). The following search parameters were used: Keywords “congenital diaphragmatic hernia”, “gene”; Publication dates: 1 January 1990 to 31 July 2017; Languages “English”. This included individual case reports and patient series from human cases of CDH, genetic mouse models describing diaphragm defects, and literature reviews. Each entry in our gene list contains a reference to the primary source used to justify the inclusion of that gene in our analysis.

### Categorization of CDH-associated genes

CDH-associated genes were categorized using several criteria to facilitate our analysis. This included the following categories: species, phenotype, and relationship to retinoid signalling. The “species” category included *human*, *mouse*, *human and mouse*, or *suspected* and was determined based on what species diaphragm defects were identified in for a given gene. The identification and confirmation of mouse and human gene orthologs was performed using the NCBI Gene search engine (https://www.ncbi.nlm.nih.gov/gene). The “phenotype’ category included *Bochdalek*, *Muscle/eventration* (including phenotypes affecting diaphragm muscularization and diaphragm eventration)*, Morgagni, central tendon, unspecified* (the exact CDH phenotype was not definitively identified in the source material), *complex* (phenotype was reported as a combination of different diaphragm defects, e.g., Bochdalek and eventration) or *suspected* (inclusion primarily based on expression pattern, however no known associated CDH phenotype; e.g., gene expressed in the developing diaphragm). As expanded upon in our discussion, we also assessed CDH-associated genes in accordance with their relationship to the retinoic acid signaling pathway. Genes that we considered to have a relationship to the retinoic acid signaling pathway were categorized as *yes*, those genes with no known association were categorized as *no evidence*, and those genes with a suspected associated were categorized as *suspected*. For those genes that had an association, or were suspected to have an association, were further categorized according to the nature of this association. These categories included *direct* relationship (genes with a known role in retinoic acid metabolism)*, indirect* (genes that encode proteins that are known to interact with other proteins in the retinoic acid signaling pathway, typically transcriptional co-regulators)*, target genes* (genes whose expression level is known to be influenced by retinoic acid signaling), or *no association* (genes with no current evidence that they are affected by or part of the retinoic acid signalling pathway).

### Gene ontology enrichment analysis

Gene ontology characterizes the relationship between genes by specifically annotating and categorizing a gene product’s molecular function (function of the gene product) and associated biological process (series of molecular functions the gene product is a part of).^17^ This approach allows for enrichment analysis of a gene set, performed in this experiment to indicate which molecular functions and biological processes were overrepresented in our list of CDH-associated genes compared to a random gene list. We used The Gene Ontology Consortium’s online tool (http://www.geneontology.org/) for the enrichment analysis of our gene list, which was completed in July 2017. Gene names from our list were copied into the Gene Ontology tool, where we selected the species (*Homo sapiens*) and specific ontology (*molecular function* and *biological process*) for our enrichment analysis. We performed several iterations of this analysis, using the entire gene list, as well as sub-lists based on each different phenotype of CDH. Note that genes associated with a complex phenotype were included in the sub-analysis associated with their specified phenotypes. Significant terms associated with the gene set are presented along with the degree of fold enrichment, a *p*-value, and a list of CDH-associated genes linked with that term. Fold enrichment is obtained through comparing the background frequency of total genes annotated to that term in the designated species to the sample frequency representing the number of genes inputted that fall under the same term. Fold enrichment of a term was designated as overrepresented compared to the background; over representation is represented as a positive fold enrichment value. Results are presented from highest to lowest fold enrichment, only results with a *p*-value < 0.05, calculated by the Mann–Whitney *U* test, were used. The *p*-value reflects the chance of observing *n* number of genes in a gene list annotated to a specific term.^18^

## Results

### Descriptive characteristics of genes associated with abnormal diaphragm development

**Fig. 1:**
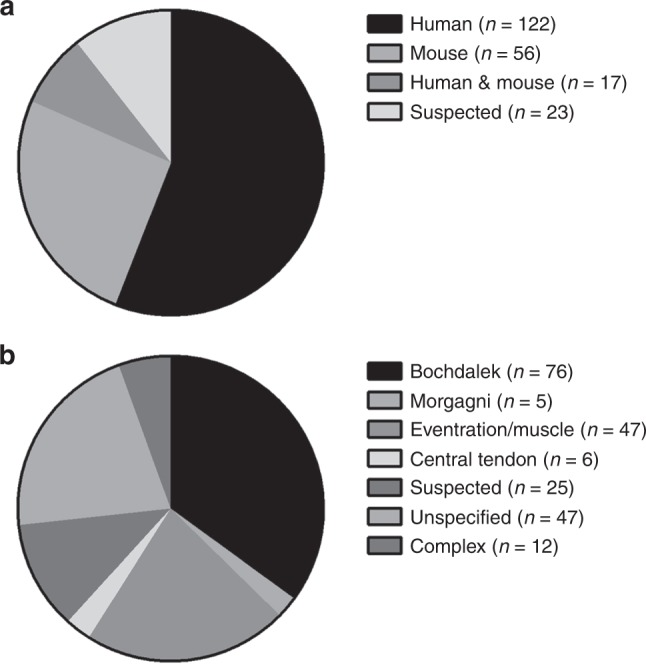
Descriptive characteristics of gene set associated with abnormal diaphragm development. Pie charts showing the relative proportion of CDH-associated genes identified in humans and mice (**a**), and the distribution of different CDH phenotypes within the gene set (**b**)

Following a comprehensive search of the literature, we identified 218 genes that are associated with diaphragm defects in humans/mice. Supplementary Table S1 (online) contains a complete list of these genes, including how they were categorized and relevant references to the literature. Some descriptive characteristics of this gene set are presented in Fig. 1. The majority of genes were identified solely from human cases of CDH (56.0%), approximately a quarter were identified from mouse phenotypes (25.7%), while less than a tenth had supporting evidence from mice and humans (7.8%; Fig. 1a). The remaining genes were included based on their suspected link to CDH (10.6%), which was primarily based on the previous literature and their known expression in the developing diaphragm (Fig. 1a). In terms of the different phenotypes of CDH, most genes were associated with Bochdalek hernias (34.9%), followed by eventration/muscle defects (21.6%), central tendon defects (2.8%), and Morgagni hernias (2.3%; Fig. 1b). A relatively large percentage of genes did not have a phenotype associated with them (33.0%), this is because their association with CDH was only suspected (11.5%), or the report describing the phenotype was not specific enough (unspecified; 21.6%).

### Enrichment analysis of all CDH-associated genes

**Fig. 2:**
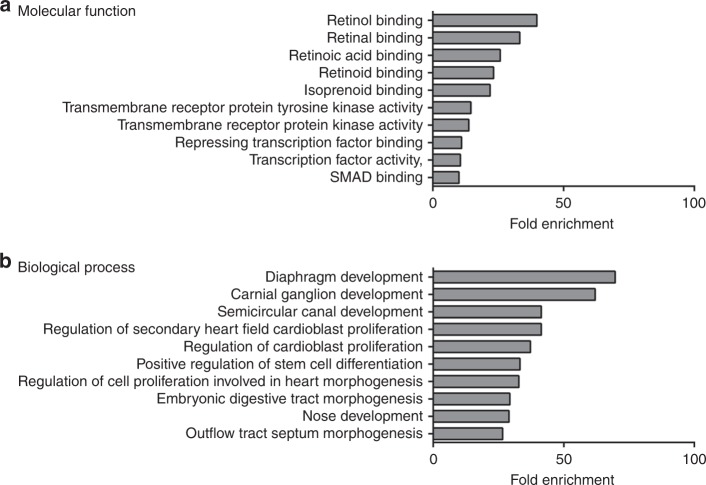
Gene ontology analysis of all CDH-associated genes. Bar charts showing the top 10 GO terms for molecular function (**a**) and biological process (**b**), ranked by fold enrichment following analysis of all CDH-associated genes

After compiling our list of CDH-associated genes, we undertook a gene ontology enrichment analysis of this gene set, processing genes in terms of their associated molecular function (Supplementary Table S2 [online]) and biological process (Supplementary Table S3 [online]). The top 10 GO terms based on molecular function and ranked by fold-enrichment are shown in Fig. 2a. The top-ranked molecular function was “Retinol binding”, and included the genes *CRABP1*, *CRABP2*, *LRAT*, *RBP1*, *RBP2*, and *RBP5*. The top 10 GO terms based on biological process and ranked by fold-enrichment are shown in Fig. 2b. The top-ranked biological process was “diaphragm development”, and included *DISP1, FGFRL1, MSC, STRA6, TCF21*, and *WT1* genes.

### Enrichment analysis of subsets of CDH-associated genes based on phenotype

**Fig. 3:**
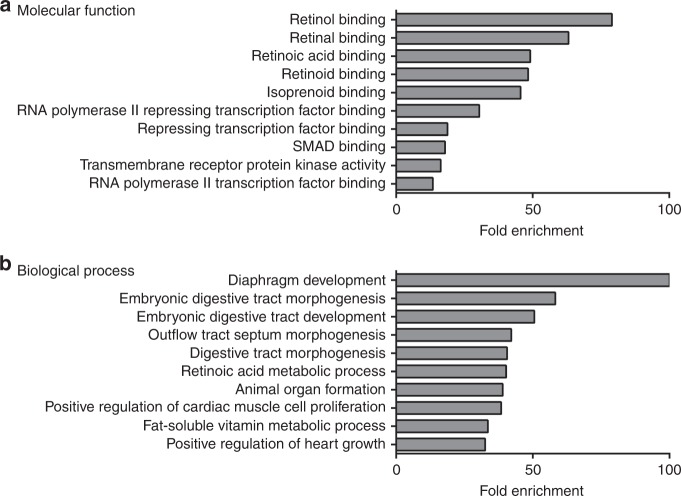
Gene ontology analysis of genes associated with Bochdalek CDH. Bar charts showing the top 10 GO terms for molecular function (**a**) and biological process (**b**), ranked by fold enrichment following analysis of genes associated with Bochdalek CDH

To determine if specific CDH phenotypes were associated with unique biological processes and molecular functions, we conducted an enrichment analysis of subsets of our gene set based on the four major CDH phenotypes: Bochdalek hernias, eventration/muscle defects, central tendon defects and Morgagni hernias. The results for our enrichment analysis of Bochdalek hernia-associated genes (76 genes) in terms of molecular function and biological process are presented in Supplementary Tables S4 and S5 (online), respectively. The top 10 GO terms based on molecular function and ranked by fold-enrichment for Bochdalek hernias are shown in Fig. 3a. Similar to the entire gene set, the top-ranked molecular function for Bochdalek hernias was “Retinol binding”, and included *CRBP1, LRAT, RBP1, RBP2*, and *RBP5* genes. The top 10 GO terms based on biological process and ranked by fold-enrichment are shown in Fig. 3b. Again, similar to the entire gene set, the top-ranked biological process was “diaphragm development”, and included *DISP1, FGFRL1, MSC, STRA6, TCF21*, and *WT1* genes.

**Fig. 4:**
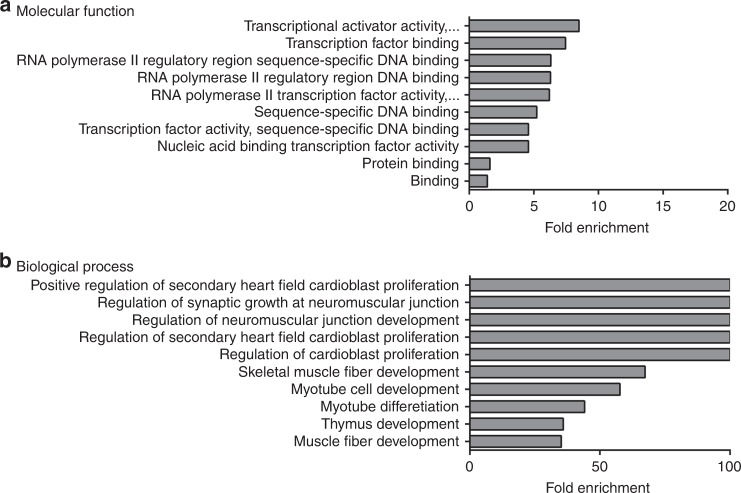
Gene ontology analysis of genes associated with diaphragm eventration and defects in diaphragm muscularization. Bar charts showing the top 10 GO terms for molecular function (**a**) and biological process (**b**), ranked by fold enrichment following analysis of genes associated with diaphragm eventration and diaphragmatic muscle defects

The results for our enrichment analysis of genes associated with diaphragm eventration/muscle defects (47 genes) in terms of molecular function and biological process are presented in Supplementary Tables S6 and S7 (online), respectively. Regarding molecular function, only 10 GO terms were significantly enriched (Fig. 4a). The top-ranked molecular function was “transcriptional activator activity”, and included *BARX2, MYOD1, MYOG, PBX1, SIX1, SIX4, SRF*, and *TBX5* genes. Indeed, as shown in Fig. 4a, many of the significantly enriched terms were related to gene transcription. The top 10 GO terms based on biological process and ranked by fold-enrichment are shown in Fig. 4b. The top-ranked biological process was “positive regulation of secondary heart field cardioblast proliferation”, and included *EYA1, SIX1*, and *TBX5* genes.

**Fig. 5:**
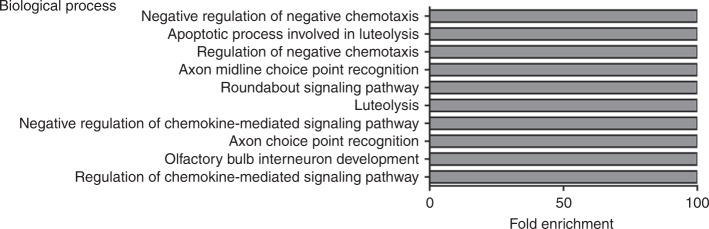
Gene ontology analysis of genes associated with central tendon defects. Bar chart showing the top 10 GO terms for biological process, ranked by fold enrichment following analysis of genes associated with diaphragmatic central tendon defects

The results for our enrichment analysis of genes associated with central tendon defects (6 genes) in terms of molecular function and biological process are presented in Supplementary Table S8 (online). There was only one GO term significantly enriched in terms of molecular function, which was “axon guidance receptor activity” and included the genes *ROBO1* and *ROBO2* (fold-enrichment >100). The top 10 GO terms based on biological process and ranked by fold-enrichment are shown in Fig. 5. The top-ranked biological process was “negative regulation of negative chemotaxis” and included *ROBO1* and *ROBO2* genes.

Likely because of the small number of genes identified in association with Morgagni hernias (5 genes), our enrichment analysis of this subset of genes did not reveal any significant terms related to molecular function of the genes, or their biological process.

### Gene association with the retinoic acid signaling pathway

As discussed below, altered retinoid acid signaling has been linked to the development of CDH.^19^ Given that our unbiased gene enrichment analysis yielded multiple GO terms associated with vitamin A metabolism and retinoic acid signaling, we re-examined our dataset to further characterize links between CDH and retinoid acid signaling. As summarized in Table 1, we were able to categorize 54 of the 218 CDH-associated genes as being linked to the retinoic acid signaling pathway. We categorized these genes as either retinoic acid target genes (44), having a direct role in retinoic acid signaling,^11^ and/or having an indirect role in retinoic acid signaling (8; Table 1). In addition to genes with a clear link, we also identified 19 genes that we suspect were linked to the retinoic acid signaling pathway though definitive evidence was not available. These suspected genes were primarily putative target genes with no strong evidence (15 genes), or genes that could be part of transcriptional protein machinery linked with retinoic acid receptors (4 genes). The identity of genes that were suspected to be linked to retinoic acid signaling are indicated in Supplementary Table S1 (online).

**Table 1:** Identity of CDH-associated genes linked to the retinoic acid signaling pathway

Direct (11)	Indirect (8)	Target gene (44)^a^
*ALDH8a1*, *CRABP1*, *CRABP2*, *LRAT*, *LRP2*, *RARA*, *RARB*, *RBP1*, *RBP2*, *RBP5*, *STRA6*	*KIF7*, *NR2F2*, *NSD1*, *RYR1*, *SIN3A*, *SRF*, *TGIF1*, *ZFPM2*	*ADD1*, *BMP4*, *CHAT*, *COL3A1*, ***CRABP1***, ***CRABP2***, *CTNNB1*, *EFNB1*, *ELN*, *FGFR2*, *FOXC1*, *GATA4*, *GATA6*, *GPRC5A*, *H19*, *HLX*, *HOXB4*, *HSD3B2*, *IGF1R*, *IGF2*, *MEIS2, MMP2*, *MNX1*, *NR2F2*, *PAX3*, *PBX1*, *PBX3*, *PDGFRA*, *PTPN13*, ***RARA***, ***RARB***, ***RBP1***, ***RBP2***, *RBPJ*, *RUNX1*, *SOX2*, ***STRA6***, *TBX6*, *TCF21*, *TGFBR3*, *WT1*, *ZEB1*, *ZFHX4*, *ZFPM2*

^a^Genes in bold were identified as target genes with a known direct role in retinoic acid metabolism, and underlined genes were identified as target genes with a known indirect role in retinoic acid metabolism

## Discussion

The goal of this study was to gain an improved understanding of the complex genetic etiology of CDH by performing a comprehensive analysis of CDH-associated genes and their relationship to defined molecular and biological processes. As highlighted by Kardon et al., with an increasing number of CDH-associated genes being identified, it is becoming important to identify links between these genes and determine if they are members of similar biological pathways important to diaphragm development.^14^ Our comprehensive review of the literature identified 218 genes that have been associated with CDH. As discussed below, our gene ontology analysis has generated unique genotype–phenotype associations between the different clinical manifestations of CDH and associated biological pathways.

Through our gene enrichment analysis of 218 CDH-associated genes, some interesting themes emerged. Importantly, as a validation of our unbiased approach, the top-ranked biological process associated with our entire gene set was *Diaphragm Development*. Given our focus on genes associated with diaphragmatic hernia, the appearance of this term at the top of our gene enrichment list while unsurprising, did highlight the utility of our approach. A dominant theme that emerged from our analysis of the molecular function of our entire gene set was links to retinoid metabolism and signaling. Indeed, the top 5 terms were directly related to retinoid metabolism, which is consistent with the hypothetical link between altered retinoic acid signaling and the development of CDH, as discussed below.^19^ While our analysis of the entire gene set generated predictable results concerning diaphragm development and retinoid metabolism, when we analyzed sub-groups of our gene set according to the different clinical manifestations of CDH we observed a unique genotype–phenotype relationship for each different type of CDH.

Of the 218 genes that we found in associated with CDH, 76 were linked with the Bochdalek type of diaphragm defects. When we analyzed this sub-group of genes the results were similar to that for the entire gene set, reflecting the fact that this group of genes made up the majority of genes we identified. Again, *Diaphragm Development* was the top biological process enriched in the set of Bochdalek CDH-associated genes. Interestingly, *Retinoic acid metabolic process* was the 6th ranked term in terms of biological process in this sub-group of genes. Consistent with this, the top 5 molecular functions enriched in the Bochdalek CDH-associated gene set were also linked to retinoid metabolism. Significantly, there were no enriched terms linked with retinoid metabolism and signaling in our sub-group analysis of other CDH phenotypes. This novel finding extends the link between retinoid acid signaling and diaphragm development and uniquely highlights the fact that altered retinoic acid signaling is a dominant driver of Bochdalek CDH, but not the other CDH phenotypes. The importance of this observation is discussed below.

Of the 218 genes that we found in associated with CDH, 47 were linked with diaphragmatic eventration and muscularization defects. When we analyzed this sub-group of genes regarding their associated biological processes two themes emerged amongst the top-ranked terms. There was a surprising link between this type of CDH and genes associated with secondary heart field development and cardioblast proliferation. Given the proximity between the developing diaphragm and the secondary heart field, this observation is perhaps unsurprising, but it does suggest that there is an unexplored link between the secondary heart field’s role in the development of the myocardium,^20^ and muscularization of the diaphragm. Another dominant theme that emerged from this analysis was terms associated with skeletal muscle differentiation and myogenesis. The results in terms of molecular function were harder to interpret and were dominated by terms linked with the control of gene transcription. A closer look at the specific genes associated with these terms in our gene set indicated a clear link with muscle development. For example, genes that recurrently appeared in association with the top-ranked terms included *MYOG* and *MYOD1*. *MYOG* and MYOD1 both encode proteins that are muscle-specific transcription factors capable of inducing a myogenic program of cell differentiation.^21^ While the preponderance of terms associated with muscle development and diaphragm eventration is perhaps unsurprising, this result does highlight an important distinction between the genotype-to-phenotype relationship of Bochdalek CDH and diaphragm eventration. Historically, it was thought that Bochdalek CDH arose from a defect in diaphragm muscularization; however, a series of seminal publications suggested that Bochdalek CDH occurs independently from myogenesis, and that it is defects in the non-muscular mesenchyme of the developing diaphragm that contribute to the development of Bochdalek hernias.^22–25^ The concept that defects in the non-muscular mesenchyme of the primordial diaphragm underlie Bochdalek CDH has been confirmed in several follow-up studies using sophisticated transgenic mouse models.^26–28^ Unlike our analysis of eventration-associated genes, GO terms linked to muscle development did not predominate our analysis of Bochdalek-associated genes. This highlights that these different types of CDH have unique genetic etiologies, and that Bochdalek CDH does not arise from mutations in genes associated with muscle development.

Of the 218 genes that we found in associated with CDH, 6 were linked with central tendon defects. Analysis of this relatively small group of genes only generated one significantly enriched molecular function (*axon-guidance receptor activity*), and 13 biological processes. In agreement with the link with axon guidance, many of the top-ranked biological processes were terms related to chemotaxis and axon guidance. The specific genes associated with these processes included *ROBO1*, *ROBO2*, and *SLIT3*. The proteins encoded by these genes form a signaling system primarily associated with axon guidance, with *ROBO1* and *ROBO2* encoding membrane receptors, which are bound by ligands from the SLIT family, including SLIT3.^29^ Consistent with the original description of their function, these genes are annotated in the GO database as being involved with axon guidance; however, in addition to this role SLIT-ROBO signaling has also been shown to have a role in many non-neuronal systems, including angiogenesis.^29^ In this context, we think that central tendon defects are not necessarily arising from issues with axon guidance, but rather aberrant angiogenesis. This proposed mechanism is supported by the recent observation that abnormal vascular development contributes to central tendon defects in mice.^30^

As indicated in the results, our analysis did not generate any significant results for the 5 genes associated with Morgagni diaphragm defects. This was likely the result of the small number of genes that we identified, and highlights the need for further genetic screening in individuals with this type of CDH in order to better understand its etiology. Interestingly, the number of genes we found in association with Morgagni diaphragm defects (2.3%) was lower than expected. This likely reflects an ascertainment bias in the diagnosis of this typically asymptomatic defect, with correspondingly less genetic screening reported in association with it.

As discussed above, genes associated with retinoid metabolism were primarily associated with Bochdalek CDH, which is consistent with the CDH retinoid hypothesis.^19^ The retinoid hypothesis states that abnormal retinoic acid signaling contributes to the development of CDH.^19^ Most of the data in support of this hypothesis has been generated from animal studies, with some supporting evidence from humans. Our study provides additional support for the retinoid hypothesis by linking CDH-associated genes with the retinoid signaling pathway. Furthermore, our analysis clearly indicates that the retinoid hypothesis applies to Bochdalek CDH, and not other CDH phenotypes, which appear to have unique genetic etiologies. As Bochdalek hernias are the most common and therefore clinically relevant presentation of CDH, we further analyzed our gene set based on possible links between a given gene and the retinoic acid signaling pathway. Several genes that were found in association with CDH have a direct role in cellular retinoic acid metabolism and signaling, further reinforcing the link between aberrant retinoic acid signaling and CDH. This includes genes encoding proteins responsible for the cellular uptake of retinol (*STRA6*, *LRP2*), cytoplasmic binding proteins of retinol and retinoic acid (*RBP1, RBP2, RBP5, CRABP1, CRABP2*), enzymes involved in cellular retinoic acid metabolism (*LRAT, ALDH8a1*), and nuclear retinoic acid receptors (*RARA, RARB*). Moreover, we identified many genes that are putative retinoic acid target genes. This suggests that the retinoic acid signaling pathway is an important regulator of diaphragm embryogenesis and that defects in this pathway, or its downstream targets, can contribute to the development of Bochdalek CDH. Continuing research in our lab is focused on understanding the link between abnormal retinoic acid signaling and Bochdalek CDH.

A limitation of this study was one inherent to the Gene Ontology database that we accessed. Following the publication of the human genome, a major initiative was started to ascribe each gene a molecular function and related biological process.^31^ This is still a work in progress, and as such there exist instances where the assignation of these attributes may not be completely accurate. As mentioned above, the annotation of specific genes to the process of axon guidance does not reflect their more recently discovered role in angiogenesis, thus making interpretation of these data more challenging. Another example is what genes the database considered to be associated with vitamin A metabolism. For example, while this process includes genes from our analysis like *LRAT* and *RBP1*, it omits other genes that have a well-established role in vitamin A metabolism, such as *RARA* and *RARB*. With this in mind, we believe that the relative importance of retinoic acid metabolism and signaling is actually underestimated in our analysis. Another limitation of our study was the large number of genes linked to an unspecified CDH phenotype (~22% of genes), which could not be included in our sub-group analysis and therefore present a lost opportunity to learn more about the genetic etiology of CDH. As recently highlighted by others,^15^ this limitation reinforces the need for accurate phenotyping of diaphragm defects. In addition to unspecified CDH phenotypes, we also identified several genes that were associated with complex phenotypes including more than one archetypal CDH phenotype. As previously commented on by Ackerman and colleagues,^15^ many clinical presentations of diaphragm defects do not fit into an archetypal phenotypic category. These complex phenotypes are difficult to interpret and are beyond the scope of this manuscript.

**Fig. 6:**
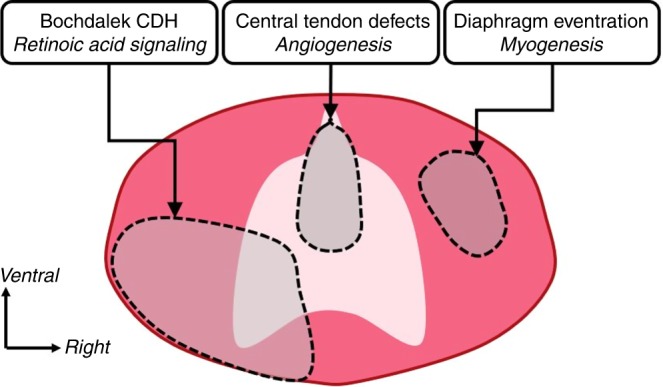
Schematic representation of human diaphragm, indicating different types of congenital diaphragmatic hernia and related pathways. This schematic shows a plan view of the diaphragm, with areas shaded in grey indicating the different types of congenital diaphragmatic hernia, as well as the pathways that contribute toward their development

In conclusion, through our analysis of a large number of CDH-associated genes we have shown that different clinical presentations of CDH have unique genetic underpinnings. Through these unique genotype–phenotype relationships, we have highlighted some of the major genetic drivers of the different types of CDH. This includes retinoic acid signaling in Bochdalek CDH, myogenesis in diaphragm eventration, and angiogenesis in central tendon defects (Fig. 6). Now that these major pathways have been identified, future work can focus on understanding the mechanistic link between specific pathways and abnormal diaphragm development. Moreover, while our discussion has largely focused on the top-ranked terms identified by our analysis, future studies can also dissect the contribution of lower ranked terms that might lead to novel insight into diaphragm development and CDH. This work will help to parse out meaning from the large number of CDH-associated genes that have been identified, establishing an important foundation for future studies focused on understanding the genotype–phenotype relationship of CDH-associated genes by identifying common pathways that contribute to abnormal diaphragm development. This is significant when evaluating the prognosis and potential future interventions for patients with specific CDH-associated gene mutations, and represents an important step toward improving the clinical outcomes of CDH.

## Electronic supplementary material

Supplementary Table

Supplementary Tables
