# Correction: Gene ontology enrichment analysis of congenital diaphragmatic hernia-associated genes

**DOI:** 10.1038/s41390-019-0536-z

**Published:** 2019-08-14

**Authors:** Timothy R. A. Dalmer, Robin D. Clugston

**Affiliations:** Department of Physiology, and Women and Children’s Health Research Institute, University of Alberta, Edmonton, AB Canada

**Correction to:** *Pediatric Research* (2019) **85**, 13–19; 10.1038/s41390-018-0192-8, published online 25 September 2018

This article was originally published under standard licence, but has now been made available under a [CC BY 4.0] license. The PDF and HTML versions of the paper have been modified accordingly.

