# Supplementary material for: Gene ontology enrichment analysis of congenital diaphragmatic hernia-associated genes
Source: Pediatr Res. 2018 Sep 25;85(1):13–9. doi: 10.1038/s41390-018-0192-8 (PMC6760551; doi:10.1038/s41390-018-0192-8)
Supplement: Supplementary file 2 — Supplementary Tables [file 41390_2018_192_MOESM2_ESM.pdf]

**Supplementary Table S2. Gene ontology analysis for entire gene set: molecular function**

| Rank | Accession number | Description | Fold enrichment | P-value | Genes |
|---|---|---|---|---|---|
| 1 | 0019841 | retinol binding | 39.83 | <0.001 | *RBP2, LRAT, RBP5, CRABP1, CRABP2, RBP1* |
| 2 | 0016918 | retinal binding | 33.19 | <0.005 | *RBP2, RBP5, CRABP1, CRABP2, RBP1* |
| 3 | 0001972 | retinoic acid binding | 25.81 | <0.01 | *NR2F2, LRAT, RBP5, CRABP1, CRABP2, RARA* |
| 4 | 0005501 | retinoid binding | 23.23 | <0.001 | *RBP2, NR2F2, LRAT, RBP5, CRABP1, CRABP2, RBP5, RBP1, RARA* |
| 5 | 0019840 | isoprenoid binding | 21.87 | <0.001 | *RBP2, NR2F2, LRAT, RBP5, CRABP1, CRABP2, RBP5, RBP1, RARA* |
| 6 | 0004714 | transmembrane receptor protein tyrosine kinase activity | 14.52 | <0.001 | *EPHB2, MET, FGFRL1, IGF1R, MUSK, EPHA3, PDGFRA, EPHA4, FGFR2, EPHA7* |
| 7 | 0019199 | transmembrane receptor protein kinase activity | 13.77 | <0.001 | *TGFBR3, LTBP4, EPHB2, MET, FGFRL1, IGF1R, MUSK, EPHA3, PDGFRA, EPHA4, FGFR2, EPHA7* |
| 8 | 0070491 | repressing transcription factor binding | 11.03 | <0.05 | *SIN3A, MKKS, CTNNB1, GATA6, CHD4, CTBP1, RBPJ* |
| 9 | 0003705 | transcription factor activity, RNA polymerase II distal enhancer sequence-specific binding | 10.54 | <0.001 | *SOX7, MYOG, GATA6, MEF2A, SRF, FOXC1, SNAI2, MSC, PBX3, FOXF2, MYOD1* |
| 10 | 0046332 | SMAD binding | 10.05 | <0.01 | *TGFBR3, TGIF1, CTNNB1, MEF2A, COL3A1, TRAP1, HMGA2, GATA4* |
| 11 | 0031490 | chromatin DNA binding | 8.99 | <0.005 | *RBP2, MYOG, SRF, CHD4, EZH2, HMGA2, MYOD1, SMARCC1, RARA* |
| 12 | 0001085 | RNA polymerase II transcription factor binding | 8.08 | <0.005 | *SIN3A, ZFPM2, MKKS, CTNNB1, GATA6, MEF2A, TBX6, CHD4, GATA4, RBPJ* |
| 13 | 0003714 | transcription corepressor activity | 6.17 | <0.001 | *TGIF1, SIN3A, HIRA, ZFPM2, CASP8AP2, NR2F2, NSD1, CTBP2, CTBP1, MSC, TBL1X, MEIS2, ZEB1, RARA, ALX1* |
| 14 | 0003682 | chromatin binding | 5.95 | <0.001 | *RBP2, UHRF1, SIN3A, HIRA, MYOG, SMARCA1, CTNNB1, SIX1, GATA6, GLI3, MEF2A, SRF, PHF21A, NIPBL, FOXC1,* |

| | | | | | |
|---|---|---|---|---|---|
| | | | | | *NSD1*, *SNAI2*, *BARX2*, *CHD4*, *CTBP2*, *GLI2*, *SMC1A*, *ZEB1*, *EZH2*, *HELLS*, *HMGA2*, *GATA4*, *CHD7*, *MYOD1*, *SMARCC1*, *RBPJ*, *RARA* |
| 15 | 0001228 | transcriptional activator activity, RNA polymerase II transcription regulatory region sequence-specific binding | 5.85 | <0.001 | *MYOG*, *WT1*, *SIX1*, *GATA6*, *GLI3*, *MEF2A*, *SRF*, *PBX1*, *FOXC1*, *TBX5*, *BARX2*, *RUNX1*, *TCF21*, *GLI2*, *MEIS2*, *SOX2*, *HMGA2*, *GATA4*, *SIX4*, *PBX3*, *FOXF2*, *MYOD1*, *RBPJ*, *ALX1* |
| 16 | 0008134 | transcription factor binding | 5.82 | <0.001 | *SIN3A*, *SMARCA1*, *ZFPM2*, *EYA2*, *MKKS*, *CTNNB1*, *SIX1*, *TWIST1*, *GATA6*, *MEF2A*, *SRF*, *PBX1*, *FOXC1*, *NSD1*, *TBX5*, *STK36*, *RUNX1*, *TBX6*, *TCF21*, *CHD4*, *CTBP2*, *GLI2*, *CTBP1*, *TBL1X*, *MEIS2*, *RARB*, *ZEB1*, *HMGA2*, *GATA4*, *FOXF2*, *MYOD1*, *RBPJ*, *ADD1*, *RARA* |
| 17 | 0001047 | core promoter binding | 5.6 | <0.05 | *RBP2*, *MYOG*, *CTNNB1*, *GATA6*, *NSD1*, *RUNX1*, *EZH2*, *HMGA2*, *MYOD1*, *RBPJ*, |
| 18 | 0004713 | protein tyrosine kinase activity | 5.53 | <0.05 | *MAP2K5*, *EPHB2*, *MET*, *FGFRL1*, *IGF1R*, *MUSK*, *EPHA3*, *PDGFRA*, *EPHA4*, *FGFR2*, *EPHA7* |
| 19 | 0044212 | transcription regulatory region DNA binding | 5.14 | <0.001 | *SOX7*, *TGIF1*, *RBP2*, *UHRF1*, *SIN3A*, *MYOG*, *WT1*, *CTNNB1*, *SIX1*, *TWIST1*, *GATA6*, *GLI3*, *MEF2A*, *SRF*, *PHF21A*, *PBX1*, *FOXC1*, *KMT2D*, *NSD1*, *TBX5*, *LONP1*, *BARX2*, *RUNX1*, *TCF21*, *CHD4*, *GLI2*, *MSC*, *TBL1X*, *MEIS2*, *SOX2*, *RBP1*, *RARB*, *ZEB1*, *EZH2*, *HMGA2*, *GATA4*, *SIX4*, *PBX3*, *CHD7*, *FOXF2*, *MYOD1*, *SMARCC1*, *RBPJ*, *RARA*, *ALX1* |
| 20 | 0000975 | regulatory region DNA binding | 5.12 | <0.001 | *SOX7*, *TGIF1*, *RBP2*, *UHRF1*, *SIN3A*, *MYOG*, *WT1*, *CTNNB1*, *SIX1*, *TWIST1*, *GATA6*, *GLI3*, *MEF2A*, *SRF*, *PHF21A*, *PBX1*, *FOXC1*, *KMT2D*, *NSD1*, *TBX5*, *LONP1*, *BARX2*, *RUNX1*, *TCF21*, *CHD4*, *GLI2*, *MSC*, *TBL1X*, *MEIS2*, *SOX2*, *RBP1*, *RARB*, *ZEB1*, *EZH2*, *HMGA2*, *GATA4*, *SIX4*, *PBX3*, *CHD7*, |

| | | | | | |
|---|---|---|---|---|---|
| | | | | | *FOXF2, MYOD1, SMARCC1, RBPJ, RARA, ALX1* |

This table presents the top 20 molecular function GO terms generated by our analysis, there were a total of 51 terms significantly associated with the entire gene set.

**Supplementary Table S3. Gene ontology analysis for entire gene set: biological process**

| Rank | Accession number | Description | Fold enrichment | P-value | Genes |
|---|---|---|---|---|---|
| 1 | 0060539 | diaphragm development | 69.7 | <0.001 | *WT1, FGFRL1, TCF21, STRA6, MSC, DISP1* |
| 2 | 0061550 | cranial ganglion development | 61.95 | <0.01 | *CTNNB1, SIX1, SEMA3A, SIX4* |
| 3 | 0060872 | semicircular canal development | 41.3 | <0.05 | *GLI3, GLI1, EYA1, CHD7* |
| 4 | 0003266 | regulation of secondary heart field cardioblast proliferation | 41.3 | <0.05 | *CTNNB1, SIX1, TBX5, EYA1* |
| 5 | 0003264 | regulation of cardioblast proliferation | 37.17 | <0.05 | *CTNNB1, SIX1, TBX5, EYA1* |
| 6 | 2000738 | positive regulation of stem cell differentiation | 33.19 | <0.005 | *HOXB4, GATA6, FOXC1, TBX5, GATA4* |
| 7 | 2000136 | regulation of cell proliferation involved in heart morphogenesis | 32.8 | <0.001 | *CTNNB1, SIX1, TBX5, BMP4, EYA1, RBPJ* |
| 8 | 0048557 | embryonic digestive tract morphogenesis | 29.35 | <0.001 | *GLI3, HLX, NIPBL, TCF21, PDGFRA, FGFR2* |
| 9 | 0043584 | nose development | 29.04 | <0.01 | *SIX1, GLI3, STRA6, SIX4, CHD7* |
| 10 | 0003148 | outflow tract septum morphogenesis | 26.55 | <0.005 | *ZFPM2, GATA6, BMP4, RARB, FGFR2, RARA* |
| 11 | 0048566 | embryonic digestive tract development | 26.55 | <0.001 | *GLI3, HLX, NIPBL, TCF21, STRA6, PDGFRA, GLI2, RARB, FGFR2, FOXF2* |
| 12 | 0072074 | kidney mesenchyme development | 25.81 | <0.05 | *WT1, SIX1, TCF21, BMP4, SIX4* |
| 13 | 0097094 | craniofacial suture morphogenesis | 25.81 | <0.05 | *MMP14, TWIST1, GLI3, BMP4, FREM1* |
| 14 | 0060045 | positive regulation of cardiac muscle cell proliferation | 24.24 | <0.005 | *TGFBR3, ZFPM2, GATA6, TBX5, FGFR2, RBPJ* |
| 15 | 0035909 | aorta morphogenesis | 24.09 | <0.001 | *SIX1, SRF, COL3A1, EYA1, CHD7, RBPJ, JAG1* |
| 16 | 0048745 | smooth muscle tissue development | 23.23 | <0.05 | *SIX1, SRF, COL3A1, STRA6, BMP4* |
| 17 | 0032331 | negative regulation of chondrocyte differentiation | 23.23 | <0.05 | *CTNNB1, SNAI2, GLI2, BMP4, RARB* |
| 18 | 0003151 | outflow tract morphogenesis | 22.87 | <0.001 | *TGFBR3, ZFPM2, SIX1, TWIST1, GATA6, NIPBL, FZD2, NEDD4, BMP4, RYR1, RARB, EYA1, FGFR2, RBPJ, RARA, JAG1* |

| | | | | | |
|---|---|---|---|---|---|
| 19 | 0061037 | negative regulation of cartilage development | 22.3 | <0.005 | *CTNNB1, SNAI2, GLI2, BMP4, RARB, RARA* |
| 20 | 0035904 | aorta development | 22.22 | <0.001 | *LOX, SIX1, SRF, COL3A1, MYH10, NDST1, EYA1, CHD7, RBPJ, LRP2, JAG1* |

This table presents the top 20 biological process GO terms generated by our analysis, there were a total of 145 terms significantly associated with the entire gene set.

**Supplementary Table S4. Gene ontology analysis for Bochdalek hernia-associated genes: molecular function**

| Rank | Accession number | Description | Fold enrichment | P-value | Genes |
|---|---|---|---|---|---|
| 1 | 0019841 | retinol binding | 78.95 | <0.001 | *RBP2, LRAT, RBP5, CRBP1, RBP1* |
| 2 | 0016918 | retinal binding | 63.16 | <0.005 | *RBP2, CRABP1, RBP5, RBP1* |
| 3 | 0001972 | retinoic acid binding | 49.13 | <0.01 | *NR2F2, LRAT, RBP5, CRABP1, RARA* |
| 4 | 0005501 | retinoid binding | 48.36 | <0.001 | *RBP2, NR2F2, LRAT, RBP5, CRABP1, RBP1, RARA* |
| 5 | 0019840 | isoprenoid binding | 45.52 | <0.001 | *RBP2, NR2F2, LRAT, RBP5, CRABP1, RBP1, RARA* |
| 6 | 0001103 | RNA polymerase II repressing transcription factor binding | 30.49 | <0.05 | *MKKS GATA6, CHD4, RBPJ* |
| 7 | 0070491 | repressing transcription factor binding | 18.74 | <0.05 | *MKKS, CTNNB1, GATA6, CHD4, RBPJ* |
| 8 | 0046332 | SMAD binding | 17.92 | <0.005 | *TGFBR3, CTNNB1M MEF2A, COL3A1, TRAP1, GATA4* |
| 9 | 0019199 | transmembrane receptor protein kinase activity | 16.38 | <0.01 | *TGFBR3, LTBP4, FGFRL1, IGF1R, PDGFRA, FGFR2* |
| 10 | 0001085 | RNA polymerase II transcription factor binding | 13.46 | <0.005 | *MKKS, CTNNB1, GATA6, MEF2A, CHD4, GATA4, RBPJ* |
| 11 | 0019838 | growth factor binding | 11.9 | <0.01 | *TGFBR3, LTBP4, COL3A1, FGFRL1, IGF1R, PDGFRA, FGFR2,* |
| 12 | 0044212 | transcription regulatory region DNA binding | 4.89 | <0.001 | *RBP2, WT1, CTNNB1, GATA6, GLI3, MEF2A, KMT2D, TCF21, CHD4, GLI2, MSC, TBL1X, SOX2, RBP1, RARB, GATA4, RBPJ, RARA* |
| 13 | 0008134 | transcription factor binding | 4.89 | <0.05 | *MKKS, CTNNB1, GATA6, MEF2A, TCF21, CHD2, GLI2, TBL1X, RARB, GATA4, RBPJ, RARA* |
| 14 | 0000975 | regulatory region DNA binding | 4.88 | <0.001 | *RBP2, WT1, CTNNB1, GATA6, GLI3, MEF2A, KMT2D, TCF21, CHD4, GLI2, MSC, TBL1X,* |

| | | | | | |
|---|---|---|---|---|---|
| | | | | | *SOX2, RBP1, RARB, GATA4, RBPJ, RARA* |
| 15 | 0001067 | regulatory region nucleic acid binding | 4.87 | <0.001 | *RBP2, WT1, CTNNB1, GATA6, GLI3, MEF2A, KMT2D, TCF21, CHD4, GLI2, MSC, TBL1X, SOX2, RBP1, RARB, GATA4, RBPJ, RARA* |
| 16 | 0000976 | transcription regulatory region sequence-specific DNA binding | 4.37 | <0.05 | *RBP2, CTNNB1, GATA6, GLI3, MEF2A, CHD4, GLI2, MSC, SOX2, RARB, GATA4, RBPJ, RARA* |
| 17 | 1990837 | sequence-specific double-stranded DNA binding | 4.14 | <0.05 | *RBP2, CTNNB1, GATA6, GLI3, MEF2A, CHD4, GLI2, MSC, SOX2, RARB, GATA4, RBPJ, RARA* |
| 18 | 0003690 | double-stranded DNA binding | 4 | <0.05 | *RBP2, WT1, CTNNB1, GATA6, GLI3, MEF2A, CHD4, GLI2, MSC, SOX2, RARB, GATA4, RBPJ, RARA* |
| 19 | 0043565 | sequence-specific DNA binding | 3.53 | <0.05 | *RBP2, WT1, CTNNB1, GATA6, GLI3, MEF2A, HLX, NR2F2, CHD4, GLI2, MSC, SOX2, RARB, GATA4, RBPJ, RARA* |
| 20 | 0003700 | transcription factor activity, sequence-specific DNA binding | 3.4 | <0.01 | *RBP2, HIRA, WT1, CTNNB1, ZXDA, GATA6, GLI3, MEF2A, NR2F2, TCF21, GLI2, MSC, SOX2, BNC1, RBP1, RARB, GATA4, RBPJ, RARA* |

This table presents the top 20 molecular function GO terms generated by our analysis, there were a total of 23 terms significantly associated with Bochdalek-associated genes.

**Supplementary Table S5. Gene ontology analysis for Bochdalek hernia-associated genes: biological process**

| Rank | Accession number | Description | Fold enrichment | P-value | Genes |
|---|---|---|---|---|---|
| 1 | 0060539 | diaphragm development | > 100 | <0.001 | *WT1, FGFRL1, TCF21, STRA6, MSC, DISP1* |
| 2 | 0048557 | embryonic digestive tract morphogenesis | 58.18 | <0.001 | *GLI3, HLX, TCF21, PGDFRA, FGFR2* |
| 3 | 0048566 | embryonic digestive tract development | 50.53 | <0.001 | *GLI3, HLX, TCF21, STRA6, PDGFRA, GLI2, RARB, FGFR2* |
| 4 | 0003148 | outflow tract septum morphogenesis | 42.11 | <0.05 | *GATA6, RARB, FGFR2, RARA* |
| 5 | 0048546 | digestive tract morphogenesis | 40.61 | <0.001 | *CTNNB1, GLI3, HLX, TCF21, STRA6, PDGFRA, GLI2, GATA4, FGFR2* |
| 6 | 0042573 | retinoic acid metabolic process | 40.2 | <0.05 | *ALDH8a1, LRAT, RBP5, CRABP1, RBP1* |
| 7 | 0048645 | animal organ formation | 39.01 | <0.001 | *CTNNB1, GATA6, GLI3, GLI2, FGFR2, RBPJ* |
| 8 | 0060045 | positive regulation of cardiac muscle cell proliferation | 38.45 | <0.05 | *TGFBR3, GATA6, FGFR2, RBPJ* |
| 9 | 0006775 | fat-soluble vitamin metabolic process | 33.5 | <0.005 | *RBP2, LRAT, RBP5, CRABP1, RBP1, LRP2* |
| 10 | 0060421 | positive regulation of heart growth | 32.51 | <0.005 | *TGFBR3, WT1, GATA6, FGFR2, RBPJ* |
| 11 | 0060411 | cardiac septum morphogenesis | 28.99 | <0.001 | *TGFBR3, GATA6, FGFRL1, RARB, GATA4, FGFR2, RARA. JAG1* |
| 12 | 0046622 | positive regulation of organ growth | 28.84 | <0.001 | *TGFBR3, WT1, GATA6, HLX, FGFR2, RBPJ* |
| 13 | 0030850 | prostate gland development | 26.32 | <0.05 | *CTNNB1, GLI3, GLI2, FGFR2, RARA* |
| 14 | 0003151 | outflow tract morphogenesis | 23.81 | <0.001 | *TGFBR3, GATA6, RARB, FGFR2, RBPJ, RARA, JAG1* |
| 15 | 0003279 | cardiac septum development | 23.52 | <0.001 | *TGFBR3, GATA6, FGFRL1, STRA6, RARB, GATA4, FGFR2, LRP2, RARA, JAG1* |
| 16 | 0003281 | ventricular septum development | 21.39 | <0.005 | *TGFBR3, FGFRL1, STRA6, GATA4, FGFR2, LRP2* |
| 17 | 0048565 | digestive tract development | 21.05 | <0.001 | *CTNNB1, GATA6, GLI3, COL3A1, HLX, TCF21, STRA6,* |

| | | | | | |
|---|---|---|---|---|---|
| | | | | | *PDGFRA*, *GLI2*, *RARB*, *GATA4*, *FGFR2* |
| 18 | 0035051 | cardiocyte differentiation | 21.05 | <0.001 | *TGFBR3*, *WT1*, *GATA6*, *MEF2A*, *PDGFRA*, *RARB*, *GATA4*, *RBPJ*, *RARA*, *JAG1* |
| 19 | 0055006 | cardiac cell development | 20.86 | <0.05 | *TGFBR3*, *MEF2A*, *PDGFRA*, *GATA4*, *JAG1* |
| 20 | 0060420 | regulation of heart growth | 20.47 | <0.05 | *TGFBR3*, *WT1*, *GATA6*, *FGFR2*, *RBPJ* |

This table presents the top 20 biological process GO terms generated by our analysis, there were a total of 108 terms significantly associated with Bochdalek-associated genes.

**Supplementary Table S6. Gene ontology analysis for diaphragm muscle defects: molecular function**

| **Rank** | **Accession number** | **Description** | **Fold enrichment** | **P-value** | **Genes** |
|---|---|---|---|---|---|
| 1 | 0001228 | transcriptional activator activity, RNA polymerase II transcription regulatory region sequence-specific binding | 8.48 | <0.05 | *MYOG, SIX1, SRF, PBX1, TBX5, BARX2, SIX4, MYOD1* |
| 2 | 0008134 | transcription factor binding | 7.44 | <0.005 | *ZFPM2, EYA2, SIX1, SRF, PBX1, NSD1, TBX5, CTBP2, CTBP1, MYOD1* |
| 3 | 0000977 | RNA polymerase II regulatory region sequence-specific DNA binding | 6.32 | <0.05 | *MYOG, SIX1, SRF, PBX1, NSD1, TBX5, BARX2, SIX4, MYOD1* |
| 4 | 0001012 | RNA polymerase II regulatory region DNA binding | 6.29 | <0.05 | *MYOG, SIX1, SRF, PBX1, NSD1, TBX5, BARX2, SIX4, MYOD1* |
| 5 | 0000981 | RNA polymerase II transcription factor activity, sequence-specific DNA binding | 6.2 | <0.005 | *MYOG, ZFPM2, SIX1, SRF, PBX1, TBX5, BARX2, PAX7, SIM2, SIX4, MYOD1* |
| 6 | 0043565 | sequence-specific DNA binding | 5.23 | <0.005 | *MNX1, MYOG, SIX1, SRF, HLX, PBX1, NSD1, TBX5, BARX2, PAX7, SIX4, PAX3, MYOD1,* |
| 7 | 0003700 | transcription factor activity, sequence-specific DNA binding | 4.58 | <0.005 | *MNX1, MYOG, ZFPM2, SIX1, SRF, PBX1, TBX5, BARX2, CTBP1, PAX7, SIM2, SIX4, PAX3, MYOD1* |
| 8 | 0001071 | nucleic acid binding transcription factor activity | 4.58 | <0.005 | *MNX1, MYOG, ZFPM2, SIX1, SRF, PBX1, TBX5, BARX2, CTBP1, PAX7, SIM2, SIX4, PAX3, MYOD1* |
| 9 | 0005515 | protein binding | 1.6 | <0.005 | *ILF3, DES, LTBP4, MMP14, MYOG, IGF2, FBN1, EFEMP2, ZFPM2, EYA2, CHRNG, SIX1, PTPRD, MMP2, SRF, HLX, PBX1, MET, FGFRL1, HADHA, NSD1, PTPRS, TBX5, ELN, TNNT3, MUSK, GAB1, DLL3, NEDD4, ADAM19, CTBP2, CTBP1, DOCK1, RYR1, DISP1, STAC3, SNAP25, SIM2, EYA1, LMNB1,* |

| | | | | | |
|---|---|---|---|---|---|
| | | | | | *PAX3, CDKN1A, MYOD1, CDKN1C* |
| 10 | 0005488 | binding | 1.41 | <0.001 | *ILF3, DES, LTBP4, MNX1, MMP14, MYOG, IGF2, FBN1, EFEMP2, EYA2, ZFPM2, CHRNG, SIX1, PTPRD, MMP2, SRF, DNASE2, HLX, PBX1, MET, FGFRL1, HADHA, NSD1, PTPRS, TBX5, ELN, TNNT3, BARX2, ECEL1, MUSK, GAB1, DLL3, NEDD4, ADAM19, CTBP2, CTBP1, DOCK1, PAX7, RYR1, DISP1, STAC3, SNAP25, SIM2, EYA1, LMNB1, SIX4, PAX3, CDKN1A, MYOD1, CDKN1C, NMNAT2* |

This table presents all of the 10 molecular function GO terms generated by our analysis of genes associated with diaphragm eventration and muscularization defects.

**Supplementary Table S7. Gene ontology analysis for diaphragm muscle defects: biological process**

| Rank | Accession number | Description | Fold enrichment | P-value | Genes |
|---|---|---|---|---|---|
| 1 | 0072513 | positive regulation of secondary heart field cardioblast proliferation | > 100 | <0.005 | *SIX1, TBX5, EYA1* |
| 2 | 0008582 | regulation of synaptic growth at neuromuscular junction | > 100 | <0.005 | *SIX1, MUSK, SIX4* |
| 3 | 1904396 | regulation of neuromuscular junction development | > 100 | <0.05 | *SIX1, MUSK, SIX4* |
| 4 | 0003266 | regulation of secondary heart field cardioblast proliferation | > 100 | <0.05 | *SIX1, TBX5, EYA1* |
| 5 | 0003264 | regulation of cardioblast proliferation | > 100 | <0.05 | *SIX1, TBX5, EYA1* |
| 6 | 0048741 | skeletal muscle fiber development | 67.31 | <0.005 | *MYOG, RYR1, STAC3, MYOD1* |
| 7 | 0014904 | myotube cell development | 57.7 | <0.01 | *MYOG, RYR1, STAC3, MYOD1* |
| 8 | 0014902 | myotube differentiation | 44.06 | <0.001 | *MYOG, BARX2, RYR1, STAC3, SIX4, MYOD1* |
| 9 | 0048538 | thymus development | 35.9 | <0.05 | *SIX1, SRF, PBX1, SIX4* |
| 10 | 0048747 | muscle fiber development | 35.12 | <0.05 | *MYOG, RYR1, STAC3, MYOD1* |
| 11 | 0060538 | skeletal muscle organ development | 33.4 | <0.001 | *MYOG, SIX1, HLX, FGFRL1, ELN, PAX7, RYR1, DISP1, STAC3, SIX4, MYOD1* |
| 12 | 0003151 | outflow tract morphogenesis | 31.07 | <0.01 | *ZFPM2, SIX1, NEDD4, RYR1, EYA1* |
| 13 | 0007519 | skeletal muscle tissue development | 28.85 | <0.001 | *MYOG, SIX1, HLX, FGFRL1, ELN, PAX7, RYR1, STAC3, SIX4, MYOD1* |
| 14 | 0014706 | striated muscle tissue development | 20.71 | <0.001 | *MYOG, EYA2, ZFPM2, SIX1, SRF, HLX, TBX5, ELN, PAX7, RYR1, STAC3, EYA1, SIX4, MYOD1* |
| 15 | 0060541 | respiratory system development | 20.5 | <0.001 | *MMP14, ZFPM2, SIX1, SRF, FGFRL1, TBX5, DISP1, SIM2, EYA1, SIX4* |
| 16 | 0016202 | regulation of striated muscle tissue development | 20.03 | <0.01 | *MYOG, ZFPM2, SIX1, TBX5, SIX4, MYOD1* |
| 17 | 0060537 | muscle tissue development | 19.77 | <0.001 | *MYOG, EYA2, ZFPM2, SIX1, SRF, HLX, TBX5, ELN,* |

| | | | | | |
|---|---|---|---|---|---|
| | | | | | *PAX7*, *RYR1*, *STAC3*, *EYA1*, *SIX4*, *MYOD1* |
| 18 | 1901861 | regulation of muscle tissue development | 19.7 | <0.01 | *MYOG*, *ZFPM2*, *SIX1*, *TBX5*, *SIX4*, *MYOD1* |
| 19 | 0048706 | embryonic skeletal system development | 19.7 | <0.01 | *MMP14*, *SIX1*, *PBX1*, *PAX7*, *EYA1*, *SIX4* |
| 20 | 0055002 | striated muscle cell development | 19.7 | <0.01 | *MYOG*, *SRF*, *RYR1*, *STAC3*, *SIX4*, *MYOD1* |

This table presents the top 20 biological process GO terms generated by our analysis, there were a total of 115 terms significantly associated with diaphragm eventration and muscularization defects.

**Supplementary Table S8. Gene ontology analysis for central tendon defects: biological process**

| Rank | Accession number | Description | Fold enrichment | P-value | Genes |
|---|---|---|---|---|---|
| 1 | 0050925 | negative regulation of negative chemotaxis | > 100 | <0.005 | *ROBO1*, *ROBO2* |
| 2 | 0061364 | apoptotic process involved in luteolysis | > 100 | <0.01 | *SLIT3*, *ROBO2* |
| 3 | 0050923 | regulation of negative chemotaxis | > 100 | <0.05 | *ROBO1*, *ROBO2* |
| 4 | 0016199 | axon midline choice point recognition | > 100 | <0.05 | *ROBO1*, *ROBO2* |
| 5 | 0035385 | Roundabout signaling pathway | > 100 | <0.001 | *SLIT3*, *ROBO1*, *ROBO2* |
| 6 | 0001554 | luteolysis | > 100 | <0.05 | *SLIT3*, *ROBO2* |
| 7 | 0070100 | negative regulation of chemokine-mediated signaling pathway | > 100 | <0.05 | *SLIT3*, *ROBO1* |
| 8 | 0016198 | axon choice point recognition | > 100 | <0.05 | *ROBO1*, *ROBO2* |
| 9 | 0021891 | olfactory bulb interneuron development | > 100 | <0.05 | *ROBO1*, *ROBO2* |
| 10 | 0070099 | regulation of chemokine-mediated signaling pathway | > 100 | <0.05 | *SLIT3*, *ROBO1* |
| 11 | 0007507 | heart development | 24.32 | <0.01 | *NDST1*, *LOX*, *GATA4*, *ROBO1*, *ROBO2* |
| 12 | 0072359 | circulatory system development | 17.35 | <0.005 | *NDST1*, *LOX*, *ROBO4*, *GATA4*, *ROBO1*, *ROBO2* |
| 13 | 0009653 | anatomical structure morphogenesis | 8.16 | <0.05 | *NDST1*, *SLIT3*, *LOX*, *ROBO4*, *GATA4*, *ROBO1*, *ROBO2* |

This table presents all of the 13 molecular function GO terms generated by our analysis of genes associated with diaphragmatic central tendon defects.
